# Ceftiofur reduced *Fusobacterium* leading to uterine microbiota alteration in dairy cows with metritis

**DOI:** 10.1186/s42523-021-00077-5

**Published:** 2021-01-28

**Authors:** Soo Jin Jeon, Federico Cunha, Rodolfo Daetz, Rodrigo C. Bicalho, Svetlana Lima, Klibs N. Galvão

**Affiliations:** 1 Department of Veterinary Biomedical Sciences, College of Veterinary Medicine, Long Island University, Brookville, NY 11548 USA; 2 Department of Large Animal Clinical Sciences, College of Veterinary Medicine, University of Florida, Gainesville, FL 32611 USA; 3 Department of Population Medicine and Diagnostic Sciences, Cornell University, Ithaca, NY 14853 USA; 4 Present Address: Department of Medicine, Weill Cornell Medicine, New York, NY 10065 USA; 5 D. H. Barron Reproductive and Perinatal Biology Research Program, University of Florida, Gainesville, FL 32611 USA

**Keywords:** Dairy cow, Metritis, Ceftiofur, Antibiotics, Uterine microbiota, *Fusobacterium necrophorum*, Metagenomics

## Abstract

**Background:**

Metritis is an inflammatory uterine disease found in ~ 20% of dairy cows after parturition and associated with uterine microbiota with high abundance of *Fusobacterium*, *Bacteroides*, and *Porphyromonas*. Ceftiofur is a common treatment, but the effect on uterine microbiota is poorly understood. Herein, we investigated the short-term impact of ceftiofur on uterine microbiota structure and function in cows with metritis. Eight cows received ceftiofur (CEF) and 10 remained untreated (CON). Uterine swabs were collected for PCR and metagenomic analysis at diagnosis before treatment (5 ± 1 DPP) and 2 days after diagnosis/treatment (7 ± 1 DPP) from the same individuals. Seven CEF and 9 CON passed quality control and were used for 16S rRNA gene sequencing.

**Results:**

Ceftiofur treatment resulted in uterine microbiota alteration, which was attributed to a decrease in relative abundance of *Fusobacterium* and in gene contents involved in lipopolysaccharide biosynthesis, whereas uterine microbiota diversity and genes involved in pantothenate and coenzyme A biosynthesis increased. Ceftiofur treatment also reduced rectal temperature and tended to reduce total bacteria in the uterus. However, other uterine pathogens such as *Bacteroides* and *Porphyromonas* remained unchanged in CEF. The *bla*_*CTX-M*_ gene was detected in 37.5% of metritic cows tested but was not affected by CEF. We found that β-hydroxybutyric acid, pyruvic acid, and L-glutamine were preferentially utilized by *Fusobacterium necrophorum* according to metabolic activity with 95 carbon sources.

**Conclusions:**

Ceftiofur treatment leads to alterations in the uterine microbiota that were mainly characterized by reductions in *Fusobacterium* and genes involved in LPS biosynthesis, which may be associated with a decrease in rectal temperature. The increase in pantothenate and coenzyme A biosynthesis indicates microbial response to metabolic stress caused by ceftiofur. Preference of *Fusobacterium* for β-hydroxybutyric acid may help to explain why this strain becomes dominant in the uterine microbiota of cows with metritis, and it also may provide a means for development of new therapies for the control of metritis in dairy cows.

**Supplementary Information:**

The online version contains supplementary material available at 10.1186/s42523-021-00077-5.

## Background

Metritis has been found in approximately 20% of dairy cows during the first 2 weeks postpartum, with the incidence ranging from 8 to > 40% in some herds [1–4]. Metritis is diagnosed based on uterine discharge with foul odor and reddish-brown color [5, 6]. Recent studies show that metritis is associated with a dysbiosis of the uterine microbiota characterized by an increase in the relative abundance of *Fusobacterium, Bacteroides*, and *Porphyromonas* [7–12]. About 40% of cows with metritis develop a fever [1, 3], which was associated with the immune response of the cow, but not with the uterine microbiota at the time of diagnosis [7, 13].

Ceftiofur, a third-generation cephalosporin with broad-spectrum activity, is the only antibiotic approved by the US Food and Drug Administration for treatment of metritis that does not require milk withdrawal, and it has been shown to decrease rectal temperature, increase cure rate, and improve milk production and fertility [14–17]. The cure rate of ceftiofur for metritis is determined by clinical resolution and has been reported to range from 67 to 85% [3, 14, 15, 18]. Despite the therapeutic benefit of ceftiofur, long-term use in food animals holds a potential risk for emergence and spread of antibiotic resistance to this important class of antibiotics [19]. For instance, treatment of metritis with ceftiofur increased the population of third-generation cephalosporin resistant *Escherichia coli* in feces up to 16 days after treatment [20]. Intrauterine pathogenic *E. coli* found in the uterus of metritic cows have shown resistance to ceftiofur and other beta-lactams because they produce extended spectrum β-lactamases (ESBL) of the CTX-M type [21]. In cattle, ESBL have been associated with plasmids carrying the *bla*_*CTX-M*_ and *bla*_*CMY-2*_ genes [21–24]. If ESBL genes are spread to uterine microbiota due to horizontal gene transfer [25], only bacteria resistant to ESBL will thrive under antibiotic pressure, which can perturb microbiota composition and possibly affect cure rates [26].

Administration of ceftiofur hydrochloride has resulted in concentrations of ceftiofur derivatives in plasma, uterine tissues and lochial fluid that exceeded the reported MIC_90_ for uterine bacteria associated with metritis [27]. After 5 days of ceftiofur treatment, the uterine microbiota structure of metritic cows significantly changed, and the relative abundance of *Fusobacterium, Bacteroides*, and *Porphyromonas* decreased in cows that achieved resolution of metritis, whereas these pathogens remained abundant in cows that failed to cure [8]. This highlights the importance of uterine microbiota for uterine health and disease. However, we still do not have a good understanding of how ceftiofur affects the uterine microbiota and how these changes eventually lead to clinical cure of metritis. In this study, we aimed to examine the immediate effect of ceftiofur on the uterine microbiota in cows with metritis by evaluating the shift in uterine microbiota structure and function. We used this approach to minimize the effect of time on the uterine microbiota [8]. We used metabolic profiling to test 95 carbon sources to characterize *Fusobacterium necrophorum*, the uterine pathogen most sensitive to ceftiofur, and we evaluated the presence of ESBL in uterine microbiota. We also evaluated changes in rectal temperature and total bacterial load after treatment. Knowledge of the effectiveness and limitations of ceftiofur as well as understanding the metabolic needs of the main uterine pathogen will contribute to the development of more effective and alternative treatments of metritis and potentially reduce our reliance on ceftiofur.

## Results

### Descriptive statistics

Descriptive statistics for cows used for sequencing is shown in Additional file 1: Table S1. There was no effect (*P* > 0.05) of treatment on risk factor for metritis, body condition score (BCS) at 4 days postpartum (DPP), blood calcium, non-esterified fatty acids (NEFA), or β-hydroxybutyric acid (BHBA) concentrations at 4 DPP, and rectal temperature (RT) at 5 ± 1 DPP. None of the cows developed mastitis before or after enrolment. There was an effect (*P* < 0.05) of parity on BHBA at 4 DPP and on RT at 5 ± 1 DPP. Multiparous cows had higher BHBA concentrations than primiparous (1.06 ± 0.1 vs. 0.73 ± 0.1 mmol/L), and primiparous had higher RT than multiparous (39.7 °C ± 0.1 vs. 39.2 °C ± 0.1). There was no effect of parity on risk factor for metritis, BCS at 4 DPP, or blood calcium and NEFA at 4 DPP.

### Ceftiofur mainly targeted *Fusobacterium*, leading to alteration of uterine microbiota structure and function

**Fig. 1:**
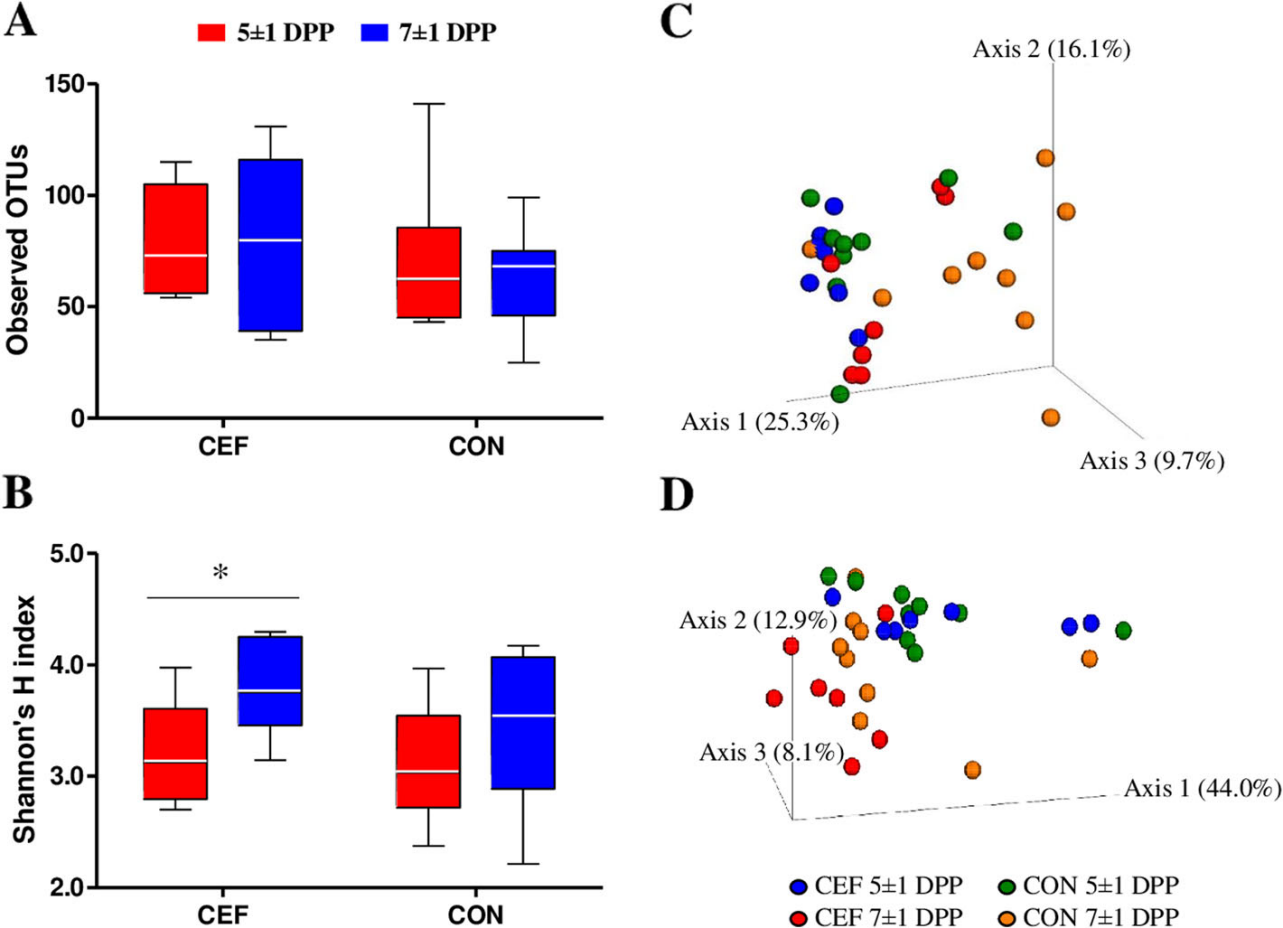
Uterine microbiota diversity. **a** Observed OTUs (CEF: *P* = 0.75, CON: *P* = 0.72). **b** Shannon’s H index (CEF: *P* = 0.05^*^, CON: *P* = 0.23). **c** Uterine microbiota on unweighted UniFrac (PERMANOVA, *P* < 0.01). **d** Uterine microbiota on weighted UniFrac (PERMANOVA, *P* = 0.01). Data in **a** and **b** present minimum, median (the line in the middle of the box), and maximum, and they were analyzed between 5 ± 1 DPP and 7 ± 1 DPP within the group using the Wilcoxon signed-rank test. Data in **c** and **d** present UniFrac distances between bacterial communities, and PERMANOVA was used to test dissimilarity between groups

**Fig. 2:**
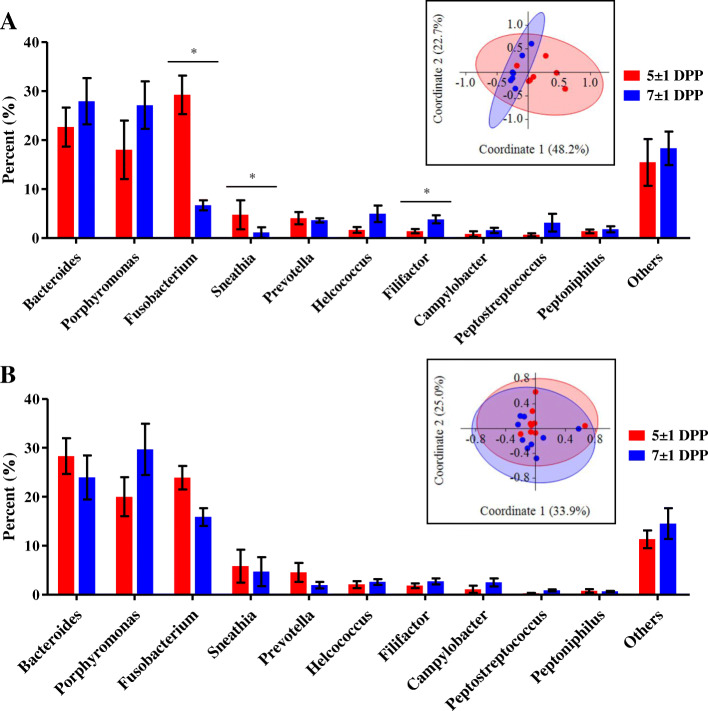
Uterine microbiota alteration. Relative abundance of bacterial genera (> 1% abundance) and PCoA based on Bray-Curtis distance of genus abundance data with 95% confidence ellipses in **a** CEF and **b** CON. Bar graphs represent mean and SEM and significant differences between 5 ± 1 DPP and 7 ± 1 DPP were analyzed using the Wilcoxon signed-rank test (*P* ≤ 0.05*). PCoA plots were used to represent the similarity of uterine microbiota, and significant difference between 5 ± 1 DPP and 7 ± 1 DPP was analyzed using PERMANOVA (CEF: *P* < 0.01, CON: *P* = 0.09)

**Fig. 3:**
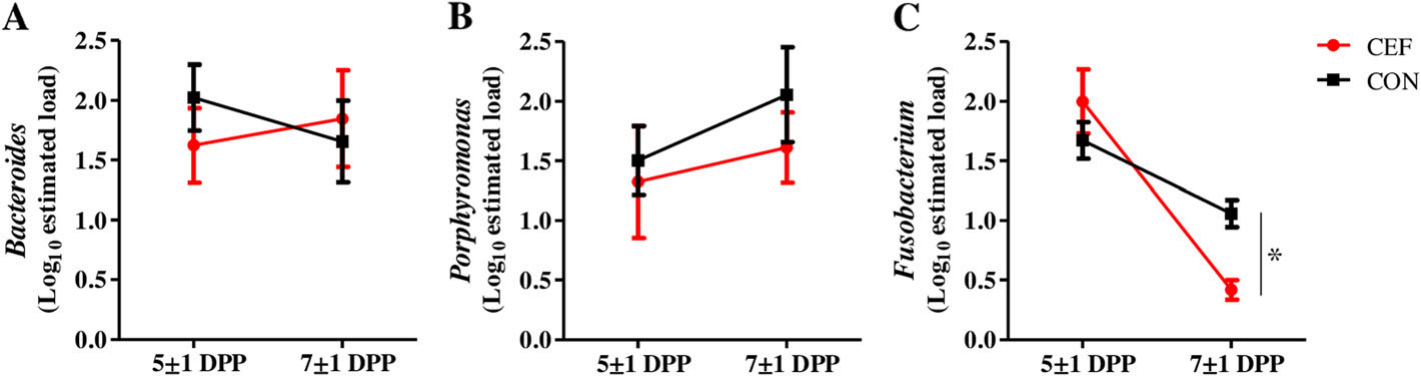
Estimated load for uterine pathogens. **a** *Bacteroides.* **b** *Porphyromonas.* **c** *Fusobacterium*. Bacterial loads were compared between groups at each time point using one-way ANOVA (*P* ≤ 0.05*). Data represent mean and SEM

**Fig. 4:**
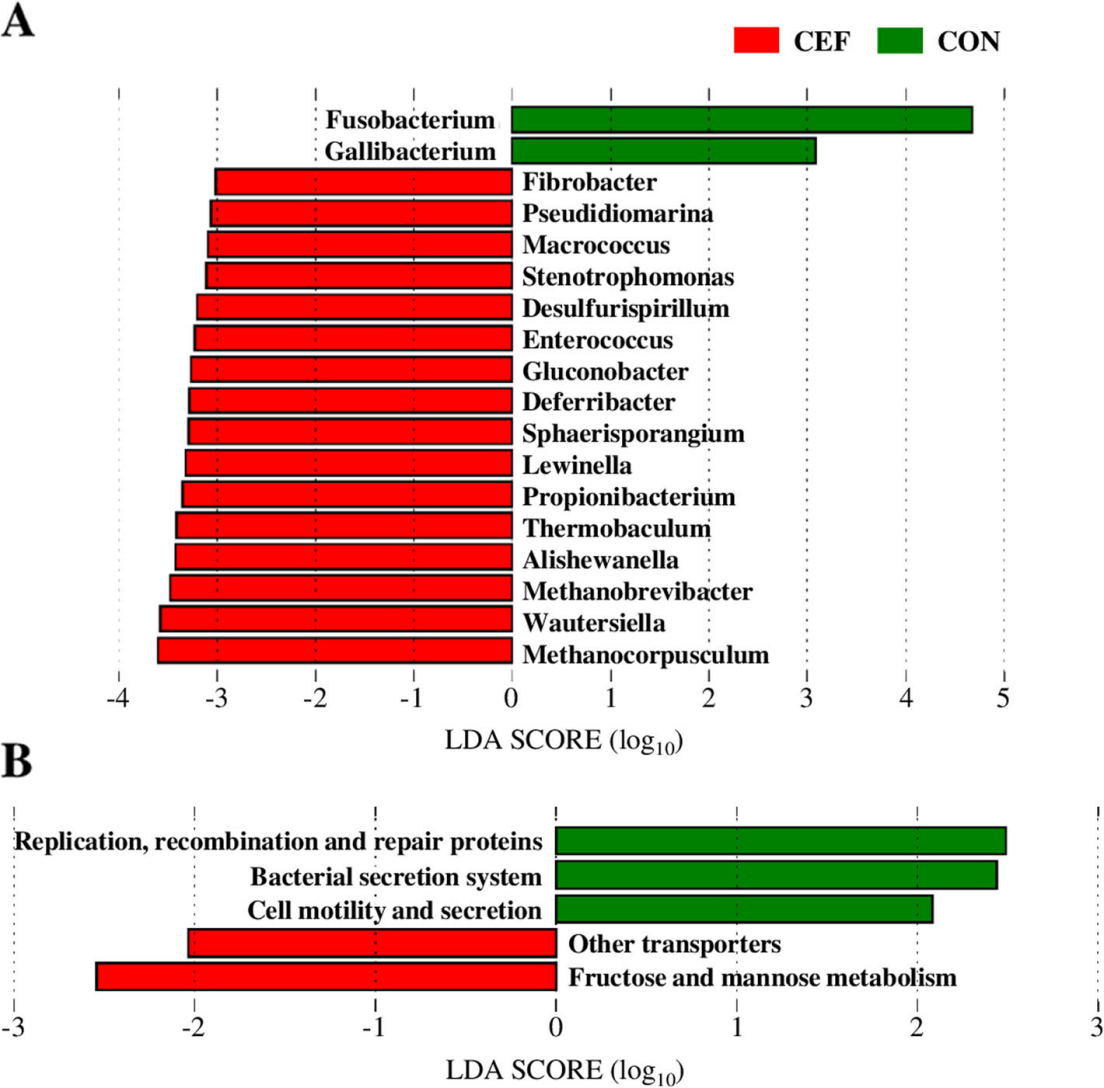
The linear discriminant analysis effect size (LEfSe) method to identify differential features between CEF and CON on 7 ± 1 DPP. **a** Taxonomic profiles with LDA score higher than 3. **b** Metabolic profiles with LDA score higher than 2

**Fig. 5:**
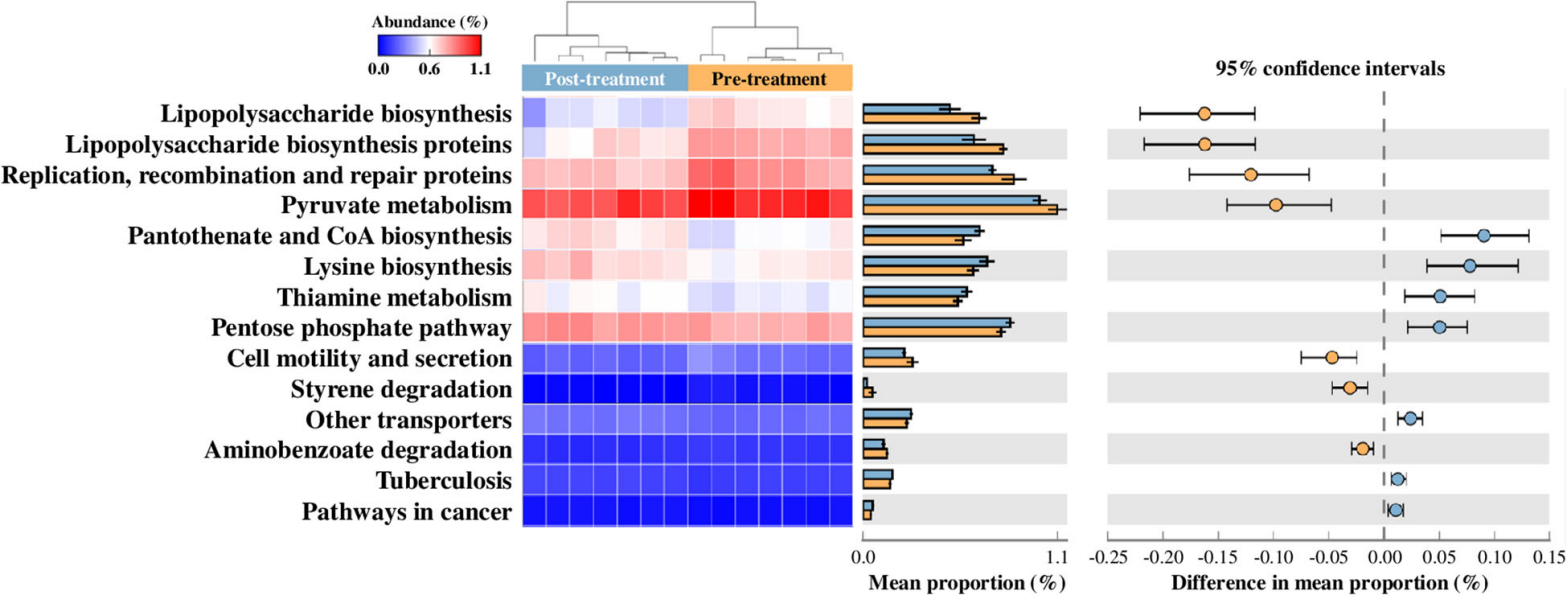
Shift in metabolic function of uterine microbiota. Heat map and extended error bar plot show the 14 gene families with statistical significance between pre- and post-treatment in CEF (White’s non-parametric t-test, two-sided, *P* < 0.01)

To evaluate the effect of ceftiofur on diversity of uterine microbiota, we examined the number of observed OTUs and Shannon’s H Index for alpha diversity and unweighted and weighted UniFrac for beta-diversity (Fig. 1). Uterine microbiota showed no significant difference in observed OTUs by treatment and time (Fig. 1a). Meanwhile, we found a significant increase (*P* = 0.05) in Shannon’s H Index in CEF following treatment from 3.2 ± 0.2 to 3.8 ± 0.2, and CON showed no significant difference (*P* = 0.26) in Shannon’s H Index from 3.1 ± 0.2 to 3.4 ± 0.2 between 5 ± 1 DPP and 7 ± 1 DPP (Fig. 1b). According to principal coordinate analysis (PCoA) plots of unweighted (Fig. 1c) and weighted UniFrac (Fig. 1d), CEF and CON had similar bacterial communities on 5 ± 1 DPP (*P* = 0.99 in Fig. 1c; *P* = 0.54 in Fig. 1d), but they showed different progressions of bacterial communities on 7 ± 1 DPP. Unweighted UniFrac distances indicated the significant difference in species composition between CEF and CON (*P* = 0.01 in Fig. 1c), and weighted UniFrac distances displayed the limited difference in abundance (*P* = 0.07 in Fig. 1d). To further identify how uterine microbiota was altered by either ceftiofur or time, we performed PCoA based on Bray-Curtis distance of genus abundance data, and we compared relative abundance of bacterial genera between 5 ± 1 DPP and 7 ± 1 DPP in each group (Fig. 2). CEF significantly changed uterine microbiota structure, which can be visualized in the PCoA plot by a shift in the uterine microbiota and an increase in homogeneity (*P* < 0.01; Fig. 2a). Comparison of relative abundances showed that there was a reduction in *Fusobacterium* (29.2% ± 3.9 vs. 6.7% ± 1.0; *P* = 0.02) and *Sneathia* (4.7% ± 3.0 vs. 1.1% ± 1.1; *P* = 0.02), as well as an increase in *Filifactor* (1.4% ± 0.4 vs. 3.8% ± 0.8; *P* = 0.02). CON displayed a tendency to change (*P* = 0.09) in the structure of the uterine microbiota, but no significant difference in relative abundance of bacterial genera between 5 ± 1 DPP and 7 ± 1 DPP (Fig. 2b). Next, we estimated bacterial loads for *Bacteroides, Porphyromonas*, and *Fusobacterium,* which are important uterine pathogens associated with metritis (Fig. 3). Estimated loads of *Bacteroides* and *Porphyromonas* remained abundant between 5 ± 1 DPP and 7 ± 1 DPP with no difference between CEF and CON. However, estimated load of *Fusobacterium* was greatly decreased in CEF on 7 ± 1 DPP when CEF was significantly lower (*P* < 0.01) than CON. To determine features that explain differences between CON and CEF on 7 ± 1 DPP, taxonomic profiles were analyzed using the LEfSe method, in which *Fusobacterium* contributed most to the differentiation between CEF and CON (Fig. 4a). Furthermore, low-abundance bacteria were an important feature of CEF, which seem to contribute to increased diversity of the uterine microbiota. Metabolic profiles were also analyzed with the LEfSe, in which CEF was characterized by high abundance of fructose and mannose metabolism and other transporters, while CON was characterized by pathways of replication, recombination and repair proteins, bacterial secretion system, and cell motility and secretion (Fig. 4b). Furthermore, the STAMP detected 14 active features that were significantly different (*P* < 0.01) between pre- and post-treatment in CEF, in which gene families involved in lipopolysaccharide (LPS) biosynthesis decreased the most after treatment (Fig. 5). CEF also increased genes involved in pantothenic acid (vitamin B5) and coenzyme A biosynthesis. Altogether, the data demonstrate that a single dose of ceftiofur resulted in alteration of uterine microbiota structure and function toward increased diversity as well as decreased *Fusobacterium* and LPS biosynthesis.

### *Fusobacterium* prefers BHBA, pyruvic acid, and L-glutamine as energy sources

**Fig. 6:**
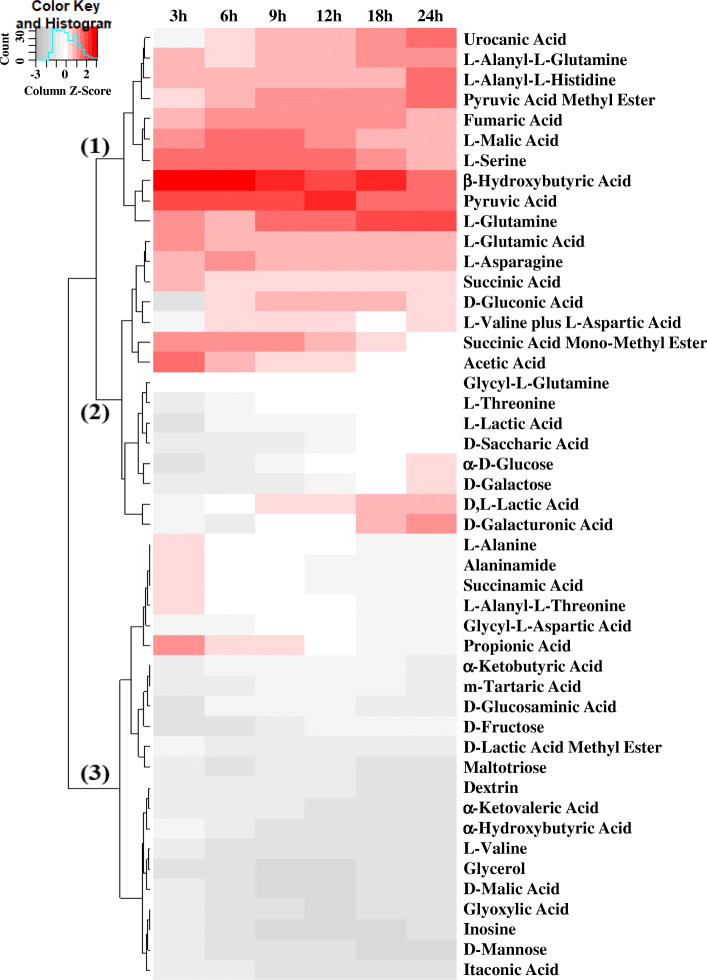
Metabolic activity of *Fusobacterium necrophorum*. *F. necrophorum* KG34 isolated from dairy cows with metritis was incubated with 95 carbon sources separately. Of them, 47 metabolized by *F. necrophorum* at 24 h post incubation are visualized in a heat map with the scale by columns

*Fusobacterium*, particularly *F. necrophorum*, was found to be the most abundant uterine pathogen in the uterus of dairy cows with metritis [9]. However, it remains unclear how *Fusobacterium* outgrows other uterine bacteria and then reduces in population over time. Assuming that bacterial growth relies on available energy sources, we examined metabolic activity of *F. necrophorum* (KG34) which was isolated from the uterus of dairy cows with metritis. Of 95 carbon sources tested, 47 carbon sources that were metabolized by KG34 at 24 h incubation were presented in a heat map (Fig. 6). These carbon sources were grouped into three clusters with different kinetic responses: the strong and fast responses to KG 34 were detected in Group 1, moderate and steady responses in Group 2, and low responses in Group 3. Particularly, KG34 metabolized BHBA, pyruvic acid, and L-glutamine most rapidly and strongly in Group 1. This may reflect energy preference for KG 34 and can be key to regulating abundance of *F. necrophorum* in the uterus.

### Ceftiofur reduced RT and tended to reduce bacterial load

**Fig. 7:**
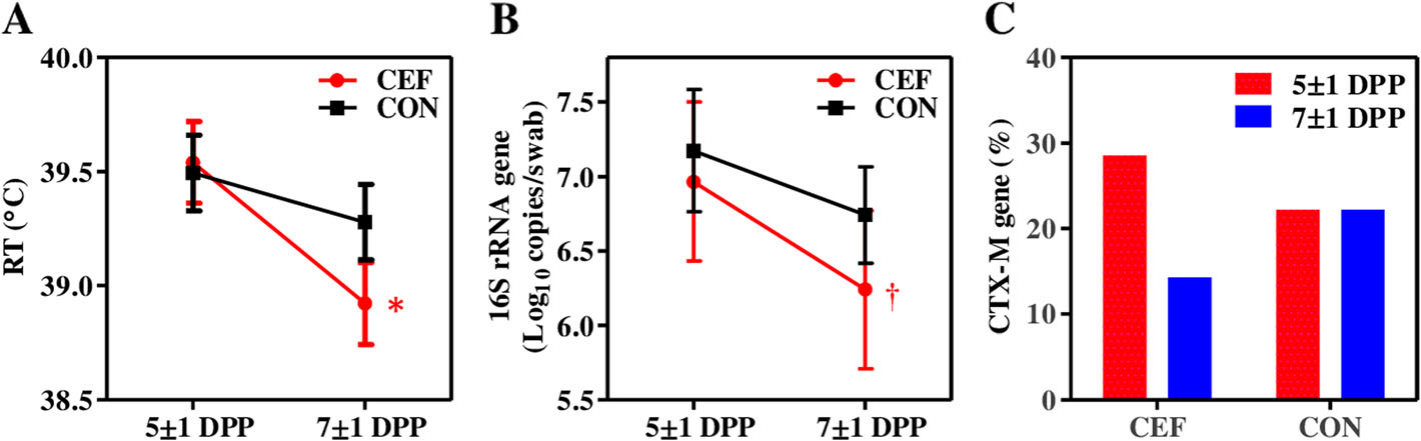
Ceftiofur effects on rectal temperature (RT), total bacteria, and antibiotic resistance in postpartum cows with metritis. **a** RT of cows was compared between 5 ± 1 DPP and 7 ± 1 DPP within the group using paired t-test (CEF: *P* = 0.04^*^, CON: *P* = 0.18). **b** The number of total bacteria in the uterus was quantified as the log copy number of the 16S rRNA gene per swab. The Wilcoxon signed-rank test was used to compare total bacteria between 5 ± 1 DPP and 7 ± 1 DPP within the group (CEF: *P* = 0.08^†^, CON: *P* = 0.30). **c** The percent of animals carrying *bla*_*CTX-M*_ gene. Data in **a** and **b** are presented by mean ± SEM

We evaluated if RT of cows with metritis was changed by a single dose of ceftiofur. There was no significant difference (*P* > 0.10) between groups at 5 ± 1 DPP or at 7 ± 1 DPP, but within the CEF group, RT significantly decreased (*P* = 0.04) from 5 ± 1 to 7 ± 1 DPP (39.5 °C ± 0.2 vs. 38.9 °C ± 0.2), and CON showed no significant change (*P* = 0.18) from 5 ± 1 to 7 ± 1 DPP (39.5 °C ± 0.2 vs. 39.3 °C ± 0.2) (Fig. 7a). Next, we quantified total bacteria using genomic DNA extracted from uterine swabs to identify if a reduction in RT was associated with a reduction in bacterial count. There was no significant difference (*P* > 0.10) between groups at 5 ± 1 DPP or at 7 ± 1 DPP, but within group, CEF showed a tendency (*P* = 0.08) for a decrease in total bacteria from 5 ± 1 to 7 ± 1 DPP (7.0 ± 0.5 vs. 6.2 ± 0.5 Log_10_ copies/swab), and CON showed no significant change (*P* = 0.30) from 5 ± 1 to 7 ± 1 DPP (7.2 ± 0.4 vs. 6.7 ± 0.3 Log_10_ copies/swab) (Fig. 7b). All together, these data indicate that a single dose of ceftiofur was capable of reducing RT and total bacteria while there was no significant change in these parameters.

### Ceftiofur did not affect the presence of ESBL genes, and ESBL genes did not affect the uterine microbiota

We examined the presence of *bla*_*CTX-M*_ and *bla*_*CMY-2*_ genes, which are responsible for resistance to extended-spectrum cephalosporins in cattle (Fig. 7c). Both groups were positive for *bla*_*CTX-M*_, with a decrease in CEF from 28.6 to 14.3% and no change in CON at 22.2% between 5 ± 1 DPP and 7 ± 1 DPP. The *bla*_*CMY-2*_ was not detected in any of the samples tested. We investigated if the presence of ESBL gene could interfere with the effect of ceftiofur on the uterine microbiota, but the presence of the *bla*_*CTX-M*_ gene in the uterus at 5 ± 1 or at 7 ± 1 DPP did not affect (*P* = 0.97) the uterine microbiota structure at 7 ± 1 DPP in the CEF group (Additional file 2: Figure S1). As expected, the presence of *bla*_*CTX-M*_ gene did not affect (*P* = 0.52) the uterine microbiota structure at 7 ± 1 DPP in the CON group (Additional file 2: Figure S1).

## Discussion

The main objective of this study was to evaluate the effect of ceftiofur on the uterine microbiota in cows with metritis. We observed that ceftiofur changed uterine microbiota structure through a significant reduction of *Fusobacterium* and *Sneathia* belonging to the phylum *Fusobacteria* and an increase in *Filifactor* belonging to the phylum *Firmicutes*. The relative abundance of *Bacteroides* and *Porphyromonas* revealed no significant change in CEF following treatment, which corroborates previous findings [8, 28, 29]. An increase in abundance of *Fusobacterium*, *Bacteroides,* and *Porphyromonas* has been shown to be associated with metritis [7, 9, 11, 12], and a reduction in these bacteria has also been associated with cure of metritis regardless of antibiotic treatment [8]. Therefore, therapeutic action of ceftiofur is likely related to a reduction in *Fusobacterium. Fusobacterium* is one of the most important and prevalent bacteria associated with the development of metritis in dairy cows [9, 30], and it appears to be involved in the change of the structure and function of the uterine microbiota associated with metritis and its cure [8]. *Fusobacterium* has been shown to increase rapidly from calving until 2 DPP [9], becomes dominant by the time of metritis diagnosis [9, 30], and remains abundant in cows that fail to cure [8]. Considering the marked improvement in clinical outcomes such as drop in RT and increase in cure rate [14, 15, 18], it is intriguing that *Fusobacterium* was the only uterine pathogen decreased in cows treated with ceftiofur. Nonetheless, in our most recent study, decreases in the phylum *Fusobacteria* and particularly the genus *Fusobacterium* appeared to be the most important contributors to resolution of metritis [31]. We observed that one of our treatments for metritis, chitosan microparticles, arrested the progression of the uterine microbiota, whereas ceftiofur-treated and untreated cows progressed towards a heathy uterine microbiota, and the most significant feature was a reduction in *Fusobacteria*/*Fusobacterium* in treated and untreated cows [31]. Therefore, we hypothesize that *Fusobacterium* and particularly *F. necrophorum* is a keystone pathogen that supports and stabilizes the dysbiotic microbiota associated with metritis [9, 13, 30, 32]. To help clarify the role of *Fusobacterium* and particularly *F. necrophorum* as a keystone pathogen, future studies should investigate whether the main uterine pathogens such as *F. necrophorum, Bacteroides pyogenes,* and *Porphromonas levii* could induce metritis, or if metritis could be cured by targeting each pathogen individually.

The rise and dominance of *Fusobacterium* is likely the result of a favorable uterine environment. To identify energy sources that may promote the growth of *Fusobacterium*, we investigated responses of *F. necrophorum* to 95 carbon sources during 24 h. Of them, 47 carbon sources were metabolized by *F. necrophorum*. This ability of *Fusobacterium* to utilize diverse metabolites may allow it to proliferate in postpartum cows using an array of energy sources. This has been observed in iron-limited conditions where *F. necrophorum* altered metabolism and increased the expression of virulence factors [33]. Nonetheless, the most important finding was the predilection of *Fusobacterium* for BHBA as an energy source. Cows undergo a period of negative energy balance around calving when energy intake cannot meet the energy demands [34–36]. This results in lipid mobilization from adipose tissue in the form of NEFA, and uptake of NEFA and partial oxidation of NEFA in the liver forms large quantities of BHBA, which end up in the blood circulation [34, 36]. More specifically, cows that develop metritis have higher NEFA and BHBA concentrations than healthy cows [37, 38], and the higher NEFA and BHBA concentrations have been shown to impair leukocyte function [38–40], which is believed to predispose to metritis. At the same time, high concentrations of BHBA may promote the proliferation of *F. necrophorum* in the uterus of dairy cows because BHBA appeared to be a preferred carbon source for *F. necrophorum*. Its preference for L-glutamine and pyruvic acid (pyruvate) seems to be of lesser relevance for its rapid growth in the uterus during early lactation because glutamine and pyruvic acid are actually decreased in early lactation due to mammary gland uptake for milk production [41–43]. Therefore, energy metabolites and their availability in the uterus seem to be significant to regulate both host immunity and uterine pathogen growth, which ultimately may determine health or disease. Further studies investigating the association between energy metabolites and growth of pathogenic uterine bacteria are warranted to develop new therapies to prevent or treat metritis.

This study shows the effectiveness and limitation of ceftiofur in eliminating uterine pathogens in dairy cows with metritis. A single dose of ceftiofur decreased *Fusobacterium* levels, but it had no effect on *Bacteroides* and *Porphyromonas* levels. Nearly all *Bacteroides* isolated from humans have been found to have resistance to third-generation cephalosporins [44, 45], and some clinical isolates of *Porphyromonas* spp. from humans have also been shown to produce β-lactamase [46, 47]; therefore, it is possible that a large proportion of *Bacteroides* and *Porphyromonas* from cattle are also resistant to ceftiofur, which needs further investigation. In addition, mechanisms of ceftiofur resistance by *Filifactor*, which increased after ceftiofur treatment also warrants further investigation.

We additionally observed a significant reduction in RT in cows treated with a single dose of ceftiofur while the same was not observed in untreated cows. This drop in RT in CEF corroborates the finding of a tendency for decreased total bacterial load and a decrease in the abundance of gene families involved in LPS biosynthesis. LPS from Gram-negative bacteria is a powerful pyrogen [48]; therefore, the decrease in LPS biosynthesis is likely related to the decrease in *Fusobacterium*, the most prevalent Gram-negative bacteria in cows with metritis [9, 31]. We also saw an increase in genes involved in pantothenic acid (vitamin B5) and coenzyme A biosynthesis. Coenzyme A (CoA) is an essential cofactor that is synthesized in a highly conserved process in prokaryotes and eukaryotes that requires pantothenic acid (vitamin B5), cysteine, and ATP. Coenzyme A and its thioester derivatives are involved in major metabolic pathways, allosteric interactions, regulation of gene expression, and in redox regulation, which is termed protein CoAlation [49]. It has been recently reported that protein CoAlation is strongly induced in response to oxidizing agents and metabolic stress in exponentially growing bacteria as a mechanism to prevent overoxidation [49]. Beta-lactam antibiotics such as ceftiofur exert its bactericidal effect by inhibiting the synthesis of the peptidoglycan layer of bacterial cell walls but they also stimulate respiration which leads to increased intracellular accumulation of reactive oxygen species (ROS) [50]. Hence, the increase in genes involved in pantothenic acid (vitamin B5) and coenzyme A biosynthesis may indicate a response to oxidative stress caused by ceftiofur.

Because of the high prevalence of ESBL-producing *E. coli* in cows with metritis [21] and the potential for horizontal gene transfer [25] to more prevalent bacteria such as *Fusobacterium*, we hypothesized that ESBL gene-carrying bacteria could thrive under antibiotic pressure. However, we did not observe any increase in ESBL genes in ceftiofur-treated cows. In fact, the proportion of cows positive for the *bla*_*CTX-M*_ gene decreased from 5 ± 1 to 7 ± 1 DPP. However, because of the short-term and small sample size in this experiment, further studies are warranted to investigate the effect of ceftiofur treatment of metritis on antimicrobial resistance in uterine bacteria.

## Conclusions

Metritis is an inflammatory disease in the uterus associated with a dysbiosis of the uterine microbiota that is characterized by high abundance of *Fusobacterium*, *Bacteroides,* and *Porphyromonas*. Ceftiofur treatment resulted in reductions in relative abundance of *Fusobacterium* and genes involved in LPS biosynthesis, whereas uterine microbiota diversity and genes involved in pantothenate and coenzyme A biosynthesis increased. We also observed a decrease in RT and a tendency for a decrease in uterine bacterial load in ceftiofur-treated cows, which corroborates the reduction of LPS biosynthesis genes, possibly related to the reduction in *Fusobacterium*. *F. necrophorum* was found to preferentially utilize BHBA, pyruvate, and L-glutamine as carbon sources. The increase in pantothenate and coenzyme A biosynthesis indicates microbial response to metabolic stress caused by ceftiofur. The relative abundance of *Bacteroides* and *Porphyromonas* and the presence of the ESBL gene were unaffected by ceftiofur treatment. In summary, ceftiofur treatment leads to alterations in the uterine microbiota mainly characterized by reductions in *Fusobacterium* and genes involved in LPS biosynthesis, which may be associated with a decrease in RT. *F. necrophorum* preference for BHBA may help to explain why this bacterium becomes dominant in the uterine microbiota of cows with metritis, and it also may provide a means for development of new therapies for the control of metritis in dairy cows.

## Methods

### Animals, treatments and sampling

Eighteen cows diagnosed with metritis at a dairy in Central Florida milking 5000 Holstein cows were used in this study. Cows were examined for clinical sign of metritis at 4 and 6 (5 ± 1) DPP, and the diagnosis was made based on the uterine discharge as previously described [5, 7, 9]. Briefly, the uterine discharge was retrieved using the Metricheck™ device (Simcro, Hamilton, New Zealand) and scored as 1 = not fetid normal lochia, viscous, clear, red, or brown; 2 = cloudy mucoid discharge with flecks of pus; 3 = not fetid mucopurulent discharge with < 50% pus; 4 = not fetid mucopurulent discharge with ≥ 50% pus; 5 = fetid red-brownish, watery discharge. Cows with a discharge score ≤ 4 were diagnosed as healthy, and cows with a score of 5 were diagnosed with metritis as previously reported [9, 15, 18]. Cows diagnosed with metritis were randomly assigned to one of two treatments without regard to parity or RT at metritis diagnosis: CEF (*n* = 8; 4 primiparous and 4 multiparous) = received 6.6 mg/kg of ceftiofur crystalline free acid (Excede®, Zoetis) via s.c. injection at the base of the ear; CON (*n* = 10; 3 primiparous and 7 multiparous) = remained as untreated controls. In our previous study [7], there was no significant difference in uterine microbiota between metritic cows with fever and no fever. Thus, presence of fever (RT ≥ 39.5 °C) was not considered in the diagnosis of metritis or the allocation of cows to the treatments. We collected uterine swabs from the cows at metritis diagnosis (5 ± 1 DPP; pre-treatment) using a 30″ double-guarded sterile culture swab (Continental Plastics Corporation, Delavan, WI) as previously performed [7, 9]. The instrument was gently passed through the cervix and positioned in the uterine body where the internal sheath and the swab were exposed, and the swab was carefully rolled against the uterine wall. The swab was retracted within the double sheath before removal from the cow. Two days after metritis diagnosis/treatment (7 ± 1 DPP; post-treatment), uterine swabs were again collected from the same 18 cows. Swabs were delivered to the laboratory on ice within 4 h and stored at − 80 °C until DNA extraction. RT was measured in all cows at 5 ± 1 and 7 ± 1 DPP from 0700 to 0900 h immediately after milking and before swab sampling using a digital thermometer (Model GLA M700) as previously described [1]. Briefly, the rectal probe was introduced in the rectum and pressed against the rectal wall, and the reading was recorded when the reading did not change for at least 5 s. Cows with RT ≥ 39.5 °C are considered to have a fever [5]. Occurrence of risk factor for metritis (i.e. dystocia, twins, stillbirth, and retained placenta) at parturition or within 24 h of parturition was recorded, BCS at 4 DPP was recorded, blood calcium, NEFA, and BHBA concentrations at 4 DPP were assayed as previously reported [4]. Cows were monitored for mastitis during the experiment but none developed mastitis.

### DNA extraction

A frozen swab was incubated in 1 mL of phosphate-buffered saline (PBS) on ice for 2 h and then vortexed vigorously to release uterine bacteria from the swab. The swab was discarded, and PBS suspension was used to isolate bacterial genomic DNA (gDNA) using the QIAamp DNA Mini kit (Qiagen) according to the manufacturer’s protocol with a minor modification of pre-incubation with lysozyme (Thermo Fisher Scientific Part No. 90082) for a final concentration of 500 μg/mL. Briefly, bacterial cells in the PBS were lysed with 500 μg of lysozyme for 1 h at 37 °C to maximize bacterial DNA extraction. Cell lysates were added with 20 μL of Proteinase K and then incubated at 56 °C for 10 min. To remove RNA, 4 μL of RNase A (100 mg/mL, Qiagen) was added and incubated for 2 min at room temperature. The lysates were mixed with 100 μL of Buffer AL and incubated at 70 °C for 10 min. Subsequently, 200 μL of 100% ethanol was mixed with the lysates. The mixture from the previous step passed through the QIAamp Mini spin column in a 2-mL tube and was washed with 500 μL of AW1 and AW2 buffer. Finally, gDNA was eluted by 50 μL of AE buffer. The purity and concentration of gDNA were evaluated by a spectrophotometer (Nanodrop 2000, Thermo Scientific).

### 16S metagenomic sequencing

The V4 hypervariable region of the bacterial 16S rRNA gene was amplified using the DNA template tagged 12-bp error-correcting Golay barcodes, 10 μM of primer 515F and 806R, 1× GoTaq Green Master Mix (Promega), and 1 mM MgCl2 in triplicate. PCR was run with an initial denaturing step at 94 °C for 3 min, followed by 35 cycles of 94 °C for 45 s, 50 °C for 1 min, 72 °C for 90 s, and a final elongation step at 72 °C for 10 min. Amplicons were purified with a QIAquick PCR Purification Kit (Qiagen) and were quantified using Qubit 3.0 Fluorometer (Thermo Scientific) to standardize the concentration. Samples that failed to amplify and their matching samples taken from the same animal were removed; thus 14 samples from CEF (*n* = 7; 4 primiparous and 3 multiparous) and 18 samples from CON (*n* = 9; 2 primiparous and 7 multiparous) were submitted for sequencing. Amplicon sequencing was performed on the Illumina MiSeq platform (Illumina Inc.) using the MiSeq reagent kit v2–300 cycles as previously reported [9].

### Analysis of sequences

Downstream analysis of sequences was performed with QIIME 22019.1 [51]. Raw sequence data were quality filtered and denoised with DADA2 [52]. The number of filtered and non-chimeric sequences were described in Additional file 3: Table S2. The sequences were aligned and positions that were highly variable were masked using the mafft program [53], which was used for phylogenetic diversity analyses such as unweighted and weighted UniFrac using FastTree [54]. Alpha-diversity metrics (number of observed OTUs and Shannon’s diversity index) and beta diversity metrics (unweighted and weighted UniFrac) were estimated using q2-diversity after samples were rarefied to 16,000 sequences per sample. Taxonomy classification was determined using the MiSeq Reporter v2.3 based on an Illumina-proprietary classification algorithm and an Illumina-curated version of the Greengenes taxonomy database. Microbial functions of uterine microbiota were predicted based on 16S rRNA genes using Phylogenetic Investigation of Communities by Reconstruction of Unobserved States (PICRUSt)-1.1.4 [55]. The taxonomic and metabolic profiles were analyzed in linear discriminant analysis effect size (LEfSe) [56] and the Statistical Analysis of Metagenomic Profiles (STAMP) v2.1.3 [57] in order to identify features that were statistically significant between groups.

### Quantification of Total Bacteria

For standard curves, DNA template was generated on the T100 thermal cycler (Bio-Rad) using *E. coli* strain isolated from the uterus of dairy cows and primers 1056F and 1456R [22]. The PCR cycling condition consisted of 94 °C for 2 min, 40 cycles of 94 °C for 1 min, 57 °C for 1 min, 68 °C for 1 min, and 68 °C for 5 min. These PCR products were purified using QIAquick PCR Purification Kit (Qiagen) and its concentrations were determined in NanoDrop 2000 spectrophotometer (Thermo Scientific) at a wavelength of 260/280. The PCR product was 10-fold serially diluted and used to create standard curves for qPCR assay. To quantify total bacteria in swab samples, qPCR analysis was carried out on the 7500 Fast system (Applied Biosystems™) using DNA templates (gDNA from swab samples and PCR products), 2x QuantiTect SYBR Green PCR Master Mix (Qiagen) and universal primers p201 and p1370 [58]. The qPCR cycling condition was 35 cycles of 94 °C for 15 s and 60 °C for 1 min. All reactions were run in duplicate and the total copy number for each swab was calculated based on the 10-fold standard curves.

### Metabolic profiling of *F. necrophorum*

We used the AN MicroPlate (Biolog) containing 95 carbon sources to determine metabolic activity of *F. necrophorum*. The assay was carried out according to the manufacturer’s instructions. Briefly, *F. necrophorum* KG34 (GenBank accession no. SRX5402669) was grown on Wilkins-Chalgren agar medium (Sigma-Aldrich) and suspended in 14 mL of AN inoculating fluid (Biolog) to make bacterial suspension with a transmittance level of about 65%. A 100 μL of bacterial suspension was quickly plated into each well of the AN MicroPlate and incubated at 37 °C in the GasPak EZ anaerobic pouch system (BD). Utilization of the carbon sources by *F. necrophorum* was indicated by tetrazolium violet forming a blue color, which was measured at 3, 6, 9, 12, 18, and 24 h post inoculation at OD590 using SmartSpec 3000 spectrophotometer (Bio-Rad).

### Statistical analysis

Only cows that were used for sequencing were used in the statistical analysis. Continuous data such as RT, 16S rRNA gene copy number, diversity indices, and relative abundance of bacteria were compared between groups using multivariable generalized linear models in JMP Pro15. The models included the effects of treatment (CEF vs. CON), parity (primiparous vs. multiparous), time (5 ± 1 vs. 7 ± 1 DPP), and interaction between treatment and time or parity and time. There was an effect (*P* < 0.05) of parity on BHBA at 4 DPP and on RT at 5 ± 1 DPP. Multiparous had higher BHBA concentrations than primiparous (1.06 ± 0.1 vs. 0.73 ± 0.1 mmol/L), and primiparous had higher RT than multiparous (39.7 °C ± 0.1 vs. 39.2 °C ± 0.1); therefore, the results presented for treatment were adjusted for parity. The BCS and the previous occurrence of a risk factor for metritis such as dystocia, twins, stillbirth, or retained placenta were compared using multivariable generalized linear models in JMP Pro15. The models included the effects of treatment, parity, and interaction between treatment and parity. There was no effect of treatment or parity on BCS or risk factor for metritis (*P* > 0.25). None of the cows developed mastitis during the experiment. Comparisons within the group between 5 ± 1 and 7 ± 1 DPP were performed using paired t-test or Wilcoxon signed-rank test in JMP Pro15. Data from qPCR were log transformed before analysis to achieve normality. Bacterial load for uterine pathogens (*Fusobacterium, Bacteroides*, and *Porphyromonas*) was calculated by multiplying the log_10_ copy number of total bacteria by relative abundance of bacteria at the genus level as previously described [59] and differences between treatment groups were analyzed at each time point using one-way ANOVA in Minitab. The effect of treatment and parity on uterine microbiota structure and shift were examined using one-way or two-way PERMANOVA with 9999 permutations based on Bray-Curtis distance in PAST version 3.25. There was no effect of parity or interaction between parity and time on the uterine microbiota (Additional file 4: Figure S2). The LEfSe was performed on Galaxy (http://huttenhower.sph.harvard.edu/galaxy/) to characterize the differences in uterine microbiota structure and function between groups. Functional profiles of uterine microbiota predicted by PICRUSt were analyzed using post-hoc plot in STAMP. Metabolic activity of *F. necrophorum* was visualized in a heat map with the option scale by column using R version 3.6.1. Differences with *P* ≤ 0.05 were considered significant.

## Supplementary Information

**Additional file 1: Table S1.** Descriptive statistics.

**Additional file 2: Figure S1.** Uterine microbiota by the presence of ESBL gene. PCoA based on Bray-Curtis distance of genus abundance data with 95% confidence ellipses was conducted to compare uterine microbiota between cows with and without the *bla*_*CTX-M*_ gene in the uterus at 5 ± 1 or at 7 ± 1 DPP (one-way PERMANOVA, *P* = 0.97 in CEF and *P* = 0.52 in CON).

**Additional file 3: Table S2.** Metadata, MG-RAST IDs, and sequence results.

**Additional file 4: Figure S2.** Uterine microbiota by parity. **a** PCoA based on Bray-Curtis distance of genus abundance data with 95% confidence ellipses was conducted to examine uterine microbiota between primiparous and multiparous cows on 5 ± 1 DPP and 7 ± 1 DPP. The effects of parity, time, and interaction between parity and time were analyzed by using two-way PERMANOVA (Time *P* < 0.001, Parity *P* = 0.16, Interaction *P* = 0.98). **b** Relative abundance of bacterial genera (> 1% abundance) between primiparous and multiparous cows on 5 ± 1 DPP and 7 ± 1 DPP. Bar graphs represent mean and SEM, and there was no significant difference (Wilcoxon rank-sum test, *P* > 0.05) in abundance of bacterial genera by parity.

## Data Availability

Metagenome sequences analyzed during the current study are available from the MG-RAST under the ID numbers (Additional file 3: Table S2). Metadata with detailed information on parity, occurrence of risk factor for metritis (i.e. dystocia, twins, stillbirth, and retained placenta), occurrence of mastitis, BCS at 4 DPP, calcium, NEFA, and BHBA concentrations at 4 DPP, and RT before and after treatment are also available in Additional file 3: Table S2.

## Abbreviations

ANOVA: Analysis of variance
BCS: Body condition score
BHBA: β-hydroxybutyric acid
CEF: Treatment group with ceftiofur hydrochloride
CON: Untreated control group
DADA2: Divisive amplicon denoising algorithm 2
DPP: Days postpartum
ESBL: Extended-spectrum β-lactamase
gDNA: Genomic deoxyribonucleic acid
KG34: *Fusobacterium necrophorum* strain isolated from dairy cows with metritis
LDA: Linear discriminant analysis
LEfSe: Linear discriminant analysis effect size
LPS: Lipopolysaccharide
NEFA: Non-esterified fatty acids
OTUs: Operational taxonomic units
PBS: Phosphate-buffered saline
PCoA: Principal coordinates analysis
PERMANOVA: Permutational multivariate analysis of variance
PICRUSt: Phylogenetic investigation of communities by reconstruction of unobserved states
QIIME 2: Quantitative insights into microbial ecology 2
qPCR: Quantitative polymerase chain reaction
RT: Rectal temperature
SEM: Standard error of mean
STAMP: Statistical analysis of metagenomic profiles
