# Supplementary material for: Ceftiofur reduced Fusobacterium leading to uterine microbiota alteration in dairy cows with metritis
Source: Anim Microbiome. 2021 Jan 28;3:15. doi: 10.1186/s42523-021-00077-5 (PMC7844903; doi:10.1186/s42523-021-00077-5)
Supplement: Supplementary file 1 — Additional file 1: Table S1. Descriptive statistics. [file 42523_2021_77_MOESM1_ESM.pdf]

Table S1. Descriptive statistics

Variable[1]	RF	BCS	Calcium	NEFA	BHBA	RT
**Treatment**						
Ceftiofur	42.8%	3.40 ± 0.1	7.74 ± 0.2	0.75 ± 0.2	0.79 ± 0.1	39.5 ± 0.1
Untreated	55.5%	3.54 ± 0.1	7.53 ± 0.2	0.91 ± 0.2	1.00 ± 0.1	39.5 ± 0.1
**Parity**						
Primiparous	50%	3.40 ± 0.1	7.55 ± 0.2	0.81 ± 0.2	0.73 ± 0.1[a]	39.7 ± 0.1[a]
Multiparous	50%	3.55 ± 0.1	7.72 ± 0.1	0.86 ± 0.2	1.06 ± 0.1[b]	39.2 ± 0.1[b]

Different superscript letters (a, b) indicate significant differences ($P < 0.05$).

[1]RF: risk factor for metritis included occurrence of one or more of the factors dystocia, twins, stillbirth, or retained placenta; BCS: body condition score at 4 days postpartum; Calcium: blood calcium in mg/dL at 4 days postpartum, NEFA: non-esterified fatty acids in mmol/L at 4 days postpartum; BHBA: β-hydroxybutyric acid in mmol/L at 4 days postpartum; RT: rectal temperature at 5 ± 1 days postpartum.
