# Supplementary figures and images for: Ceftiofur reduced Fusobacterium leading to uterine microbiota alteration in dairy cows with metritis
Source: Anim Microbiome. 2021 Jan 28;3:15. doi: 10.1186/s42523-021-00077-5 (PMC7844903; doi:10.1186/s42523-021-00077-5)

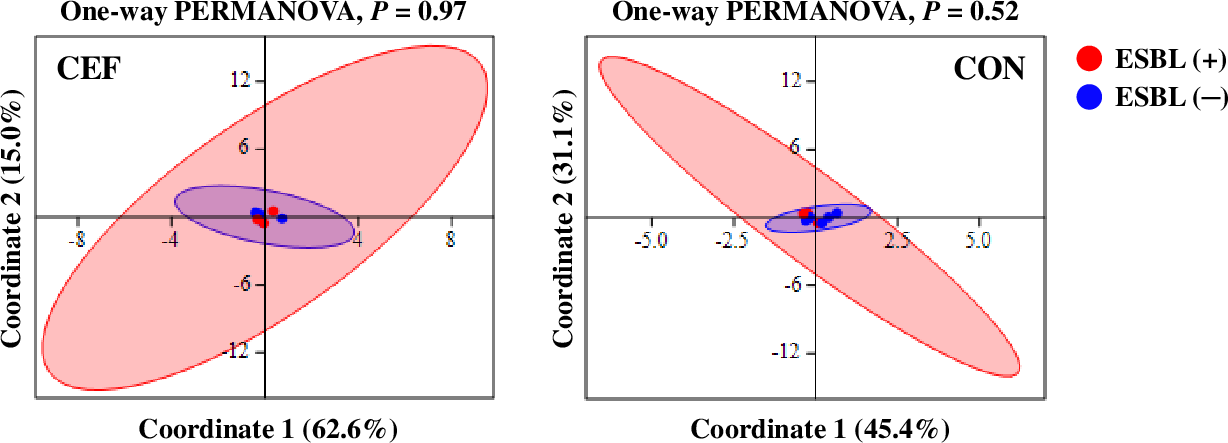

Supplement: Supplementary file 2 — Additional file 2: Figure S1. Uterine microbiota by the presence of ESBL gene. PCoA based on Bray-Curtis distance of genus abundance data with 95% confidence ellipses was conducted to compare uterine microbiota between cows with and without the blaCTX-M gene in the uterus at 5 ± 1 or at 7 ± 1 DPP (one-way PERMANOVA, P = 0.97 in CEF and P = 0.52 in CON). [file 42523_2021_77_MOESM2_ESM.tif]

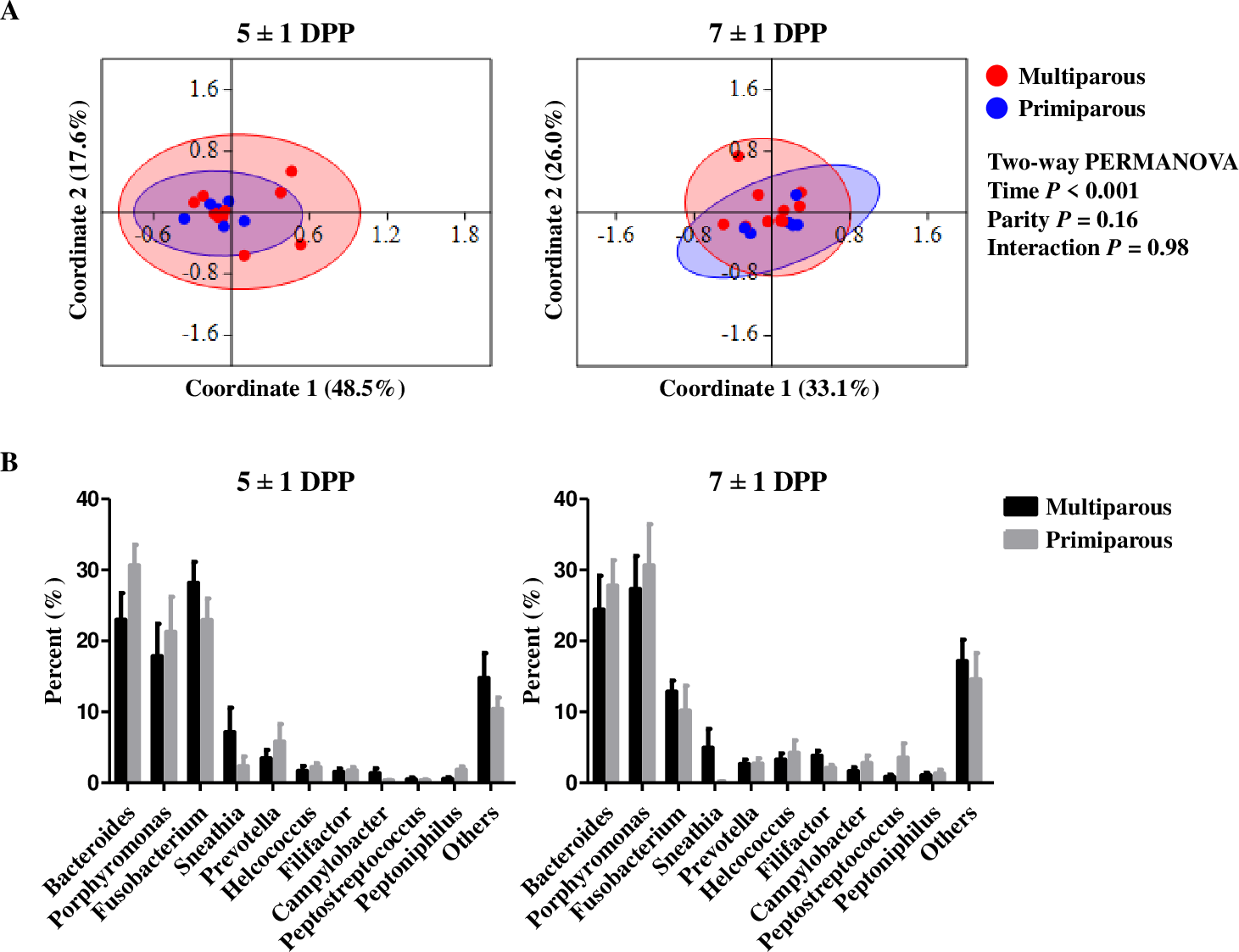

Supplement: Supplementary file 4 — Additional file 4: Figure S2. Uterine microbiota by parity. a PCoA based on Bray-Curtis distance of genus abundance data with 95% confidence ellipses was conducted to examine uterine microbiota between primiparous and multiparous cows on 5 ± 1 DPP and 7 ± 1 DPP. The effects of parity, time, and interaction between parity and time were analyzed by using two-way PERMANOVA (Time P < 0.001, Parity P = 0.16, Interaction P = 0.98). b Relative abundance of bacterial genera (> 1% abundance) between primiparous and multiparous cows on 5 ± 1 DPP and 7 ± 1 DPP. Bar graphs represent mean and SEM, and there was no significant difference (Wilcoxon rank-sum test, P > 0.05) in abundance of bacterial genera by parity. [file 42523_2021_77_MOESM4_ESM.tif]
